# OPCML, a novel systems regulator of tyrosine kinase signaling in ovarian and other cancers

**DOI:** 10.1186/1897-4287-10-S2-A34

**Published:** 2012-04-12

**Authors:** H Gabra

**Affiliations:** 1 Ovarian Cancer Action Research Centre, Department of Surgery and Cancer, Imperial College London, London, UK

## 

OPCML, a GPI anchored tumor suppressor gene is inactivated by somatic methylation in multiple cancers. We previously identified this gene by LOH mapping and demonstrated that it was inactivated by somatic methylation in 80% of ovarian cancers. Restoring OPCML expression by stable transfection suppressed in-vitro growth and in-vivo tumorigenicity. We hypothesized that OPCML might abrogate growth signaling pathways. In SKOV-3 and PEO1, ovarian cancer cell lines with no expression of OPCML, we demonstrated that OPCML negatively regulates a specific repertoire of receptor tyrosine kinases (RTKs) EPHA2, FGFR1, FGFR3, HER2 and HER4, and reciprocally, OPCML siRNA and shRNA knockdown in normal ovarian surface epithelial cells up-regulates these same RTKs, with no effect on RTKs EPHA10, FGFR2, FGFR4, EGFR, HER3, VEGFR1 and VEGFR3. shRNA knockdown shows that loss of OPCML accelerates the growth of normal ovarian surface epithelial cells in vitro. Example immunoprecipitation experiments revealed that OPCML binds to EphA2, FGFR1 and HER2 extracellular domains with no such interaction to EGFR, thus OPCML binds directly to RTKS that it negatively regulates. Cotransfection of ECD less and ECD containing Her2/Neu shows that the functional tumor suppressor phenotype of OPCML is mediated via interaction with the ECD. We demonstrate that OPCML is located exclusively in the raft membrane fraction and sequesters RTKs that it binds to the raft fraction, leading to polyubiquitination and proteosomal degradation via a cav-1 endosomal mechanism resulting in systems depletion of this specific RTK repertoire, that does not occur with RTKs that OPCML does not bind. Pulse-chase experiments confirm rapid loss of HER2 in OPCML expressing cells and not in OPCML deficient cells. We demonstrate that OPCML abrogates EGF mediated phosphorylation of FGFR1, HER2 and EGFR and the downstream phosphosignaling of pErk and pAKT. A recombinant modified OPCML-like protein without a GPI anchor, signal peptide or glycosylation was constructed and expressed in *E. coli*. This rOPCML tumor suppressor protein therapeutic caused growth inhibition by apoptosis in 6/7 ovarian cancer cell lines tested with no effect on OPCML expressing normal ovarian surface epithelium by an identical mechanism to the transfected normal protein. pERk and pAKT inhibition was seen with functional growth inhibition and increased apoptosis in rOPCML treated cells. rOPCML was then injected intraperitoneally twice weekly in two murine intraperitoneal models of ovarian cancer (nude mouse A2780 and SKOV3) and demonstrated profound inhibition of tumour weight, ascites volume and peritoneal dissemination compared with BSA control. Western analysis from rescued tumors confirmed that the same mechanism of specific RTK downregulation was also evident in vivo. In Summary, the OPCML tumour suppressor mediates its suppressor function by systems level negative regulation of at least 5 RTKs and a recombinant modified derivative is a potent tumor suppressor protein therapeutic in-vitro and in-vivo that recapitulates the in-vitro mechanism.

